# Population Genetics of Small Hive Beetles in Their Native Range Indicates High Gene Flow and Male‐Biased Dispersal

**DOI:** 10.1002/ece3.74668

**Published:** 2026-11-10

**Authors:** Michael N. K. Muturi, H. Michael G. Lattorff, Nkoba Kiatoko, Peter Neumann, Alexis Beaurepaire

**Affiliations:** ^1^ Vetsuisse Faculty, Institute of Bee Health University of Bern Bern Switzerland; ^2^ School of Agriculture and Science University of KwaZulu‐Natal Durban South Africa; ^3^ International Centre of Insect Physiology and Ecology (icipe) Nairobi Kenya; ^4^ Swiss Bee Research Centre Agroscope Bern Switzerland

**Keywords:** gene flow, genetic differentiation, genetic diversity, invasive species, sex‐biased dispersal

## Abstract

The success of biological invasions depends strongly on the genetic diversity and dispersal abilities of invading populations. In many species, males and females differ in their dispersal behavior, potentially contributing unequally to the establishment and spread of invasive populations. Population genetic approaches provide powerful tools for investigating genetic diversity and dispersal patterns. When applied to populations in their native range, these approaches can improve our ability to predict the spread and potential impacts of species following their introduction into new environments. However, such studies remain scarce for many invasive species. This holds true for small hive beetles (SHB), *Aethina tumida* , which are free‐flying parasites of social bee colonies native to sub‐Saharan Africa that have recently become a widespread invasive species. Here, we show high gene flow across *A. tumida* populations from Western Kenya. Mitochondrial DNA (mtDNA) and nuclear DNA (nuDNA) markers were used to study SHB genetic diversity and differentiation and to infer short‐ and medium‐range dispersal across host colonies (*N* = 15) and neighboring apiaries (*N* = 5; ≤ 30 km apart). The mtDNA diversity was high (29 haplotypes in 87 samples), but no significant differences were detected in haplotype diversity and distribution within and across apiaries. The nuDNA diversity and differentiation levels also did not differ significantly between apiaries. However, genetic differentiation among apiaries was significantly lower in males than in females. Moreover, significant isolation by distance was detected in the females but not in the males, suggesting sex‐biased dispersal. Overall, the results suggest that the observed high gene flow underlies the high genetic diversity of SHB, which may contribute to its invasion success.

## Graphical abstract

Small hive beetles (SHB) are honeybee pests native to SubSaharan Africa. Here, we show high gene flow in SHB populations both across and within colonies and neighboring apiaries (*N* = 5; ≤ 30 km apart) in the native range. The high gene flow observed underlies the high genetic diversity of SHB, which may contribute to its invasion success.

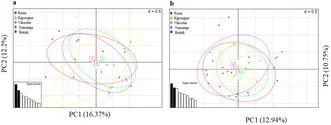

## 1 Introduction

Biological invasions are an increasing threat to biodiversity conservation, human health, and food security (Sorte 2016; Vantarová et al. 2023). Examining dispersal patterns of invasive species is thus important for identifying the factors driving their spread. However, assessing dispersal using direct estimates in the field such as mark‐recapture methods can be challenging, especially in inconspicuous species such as insects. Population genetics offers a practical alternative to these methods as it relies on indirect estimates and measures the effective dispersal which accounts for both dispersing individuals and those that successfully reproduce in their new location (Bradburd and Ralph 2019; Beaurepaire et al. 2024). It is a powerful discipline for estimating dispersal patterns over various geographical scales through genetic structure analyses and to assess and compare genetic diversity levels between and within populations (Manel et al. 2003; Kim and Sappington 2013). Given that genetic diversity levels of invasive populations are usually reduced due to bottleneck events (Nei et al. 1975), detecting fine‐scale differences in their population structure can be challenging, even when using high‐resolution markers (Dufresnes et al. 2024). Investigating the population genetics of invasive species in their native range can be used to overcome this challenge. Such analyses enable sufficient resolution for delineating populations and can help predict their spread into new areas (Beaurepaire et al. 2024).

Small hive beetles (SHB), ( *Aethina tumida* ) are parasites of bee nests native to sub‐Saharan Africa and have become invasive pests worldwide since 1996 (Lundie 1940; Neumann and Elzen 2004; Neumann et al. 2016; Papach et al. 2023). The larval stage of SHB significantly damages brood, pollen, and honey stores, eventually leading to the collapse of the entire honeybee colony (Neumann et al. 2016). In the native range, SHB is considered a minor pest (Lundie 1940; Schmolke 1974; Pirk and Yusuf 2015), while in the invasive ranges, it causes far greater damage to apiculture and possibly to wild bee populations (Neumann and Elzen 2004).

Despite these important damages, the spread of SHB remains poorly understood, yet its invasive range continues to expand (Kumaranag et al. 2025). Examining the dispersal of SHB in the native range is important as it can reveal fundamental aspects of their dispersal that contribute to their success in the invasive range (Zhang et al. 2023). Furthermore, comparing SHB dispersal between the native and invasive range may help to detect the mechanisms underlying gene flow in SHB. These mechanisms may include (i) the relative importance of migratory beekeeping (Neumann and Elzen 2004) and (ii) the high mobility of the African honeybee hosts vs. the rather stationary European hosts (Hepburn and Radloff 1998). Screening of infested and non‐infested colonies (Spiewok et al. 2008) and mark‐release‐recapture methods (Cornelissen et al. 2024) have been used to track SHB dispersal. These two direct measures of dispersal reported the natural flight capability of SHB (≤ 12 km) and frequent reinfestation within apiaries. However, these two methods do not provide information on the effective dispersal of SHB.

To date, only a few studies have used population genetics to assess the effective dispersal of SHB in its native range. Information on SHB gene flow patterns at fine spatial scales, both over short distances within apiaries and medium distances among apiaries, remains lacking. Indeed, previous studies have investigated gene flow patterns over regional and continental scales (Evans et al. 2000), showing that the spread of SHB over long distances is facilitated by migratory beekeeping and bee wax trade (Neumann and Elzen 2004; Idrissou et al. 2019). Investigating SHB movement across colonies and apiaries is essential for understanding the early stages of post‐invasion dispersal and for guiding effective mitigation strategies.

Moreover, there is a lack of genetic information on whether the dispersal patterns between SHB males and females are similar or sex‐biased. The dispersal patterns between SHB males and females may differ due to their morphological difference (e.g., females are larger than males, Sheridan et al. 2023) and behavioral differences (e.g., males tend to fly earlier than females in observational hives, Elzen et al. 2000). Assessing potential differences in dispersal between SHB sexes could help develop control measures targeting the primary disperser. For instance, understanding the dispersal patterns of male and female mosquitoes, *Aedes aegypti* , resulted in the development of an effective vector control method based on the release of sterile male mosquitoes (de Castro Poncio et al. 2023).

To shed light on these knowledge gaps, we investigated the genetic diversity levels and gene flow patterns of SHB across and within apiaries and tested if males and females SHB exhibit equal or sex‐biased dispersal patterns. To do so, male and female SHB samples were collected from five neighboring apiaries (≤ 30 km) within the native range in Western Kenya. Both mtDNA and nuDNA markers were used to characterize the genetic diversity levels and genetic structure of SHB across apiaries and colonies, as well as to infer dispersal patterns between sexes. Our results indicate a high gene flow in the region considered, and suggest contrasting patterns of dispersal between sexes, both within and across apiaries.

## 2 Material and Methods

### 2.1 Sampling

In February 2024, during the local dry season, male and female adult SHB were collected from 15 naturally infested local honeybee colonies, *Apis mellifera scutellata* (Gikungu et al. 2011), at five apiaries in Western Kenya (Figure 1 and Table 1), using standard methods (Neumann et al. 2013). All colonies were queenright and housed in Langstroth hives. The beetles were sexed in the field (Neumann et al. 2013) and preserved in 90% EtOH at −20°C until molecular analyses.

**FIGURE 1:**
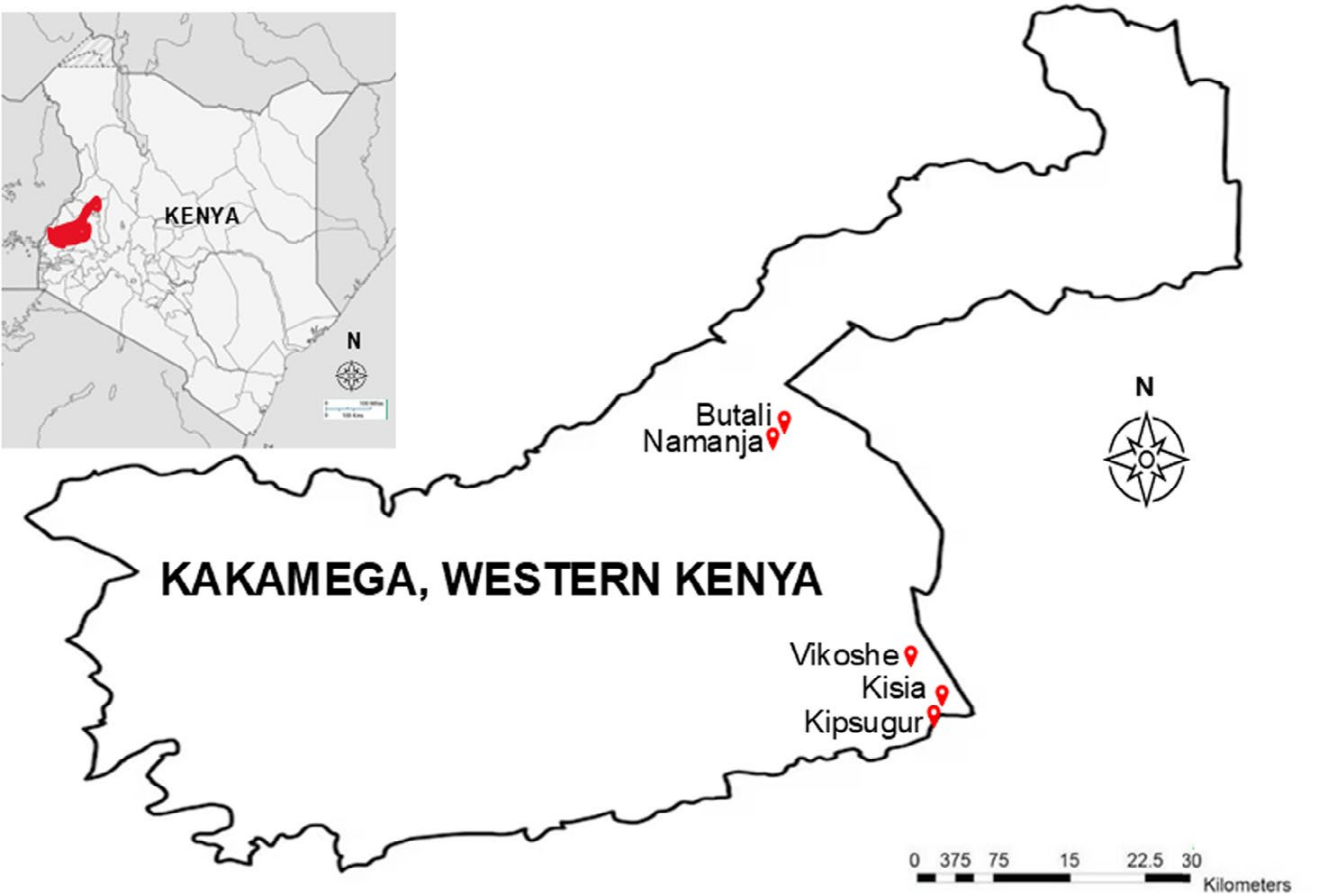
Sampling sites. *Top left*: Map of Kenya showing the sampling region (red). *Main picture*: Map of Kakamega County, Western Kenya showing the five apiaries (red symbols) from which adult small hive beetles, *Aethina tumida* , were sampled (three *Apis mellifera scutellata* colonies in each): Kisia (0.243° N, 34.963 E), Kipsugur (0.231° N, 34.952° E), Vikoshe (0.264° N, 34.938° E), Namanja (0.483° N, 34.825° E), and Butali (0.496° N, 34.835° E).

**TABLE 1:** Apiary site information, mitochondrial and nuclear DNA diversity indices.

Apiary	Colonies	Sex	*N*	*h*	*H*_*d*_	*P*_*i*_	*K*	*N*_*A*_	*H*_*O*_
Kisia	3	M	8	7	0.964 ± 0.077	0.007 ± 0.004	5.633 ± 3.028	2.875 ± 0.295	0.057 ± 0.042
		F	9	7	0.944 ± 0.07	0.007 ± 0.004	5.778 ± 3.052	4.5 ± 0.535	0.448 ± 0.106
		M + F	17	13	0.963 ± 0.078	0.007 ± 0.033	5.588 ± 2.823	3.688 ± 0.362	0.253 ± 0.075
Kipsugur	3	M	9	5	0.806 ± 0.12	0.005 ± 0.002	3.667 ± 2.046	2.875 ± 0.441	0.042 ± 0.029
		F	9	7	0.944 ± 0.07	0.006 ± 0.004	2.444 ± 1.457	4 ± 0.535	0.472 ± 0.058
		M + F	18	10	0.902 ± 0.05	0.005 ± 0.003	2.32 ± 1.77	4.25 ± 0.491	0.257 ± 0.064
Vikoshe	3	M	9	8	0.972 ± 0.064	0.006 ± 0.003	4.611 ± 2.497	3.5 ± 0.567	0.076 ± 0.062
		F	9	8	0.972 ± 0.064	0.006 ± 0.003	4.889 ± 2.489	3.75 ± 0.453	0.451 ± 0.076
		M + F	18	14	0.967 ± 0.064	0.006 ± 0.003	4.784 ± 2.452	3.625 ± 0.352	0.264 ± 0.068
Namanja	3	M	9	7	0.944 ± 0.07	0.009 ± 0.004	6.722 ± 3.501	3.125 ± 0.441	0.132 ± 0.087
		F	8	8	1 ± 0.063	0.008 ± 0.005	6.071 ± 3.234	3.625 ± 0.532	0.225 ± 0.059
		M + F	17	14	0.978 ± 0.027	0.008 ± 0.004	6.309 ± 3.302	3.375 ± 0.34	0.227 ± 0.061
Butali	3	M	8	8	1 ± 0.063	0.022 ± 0.012	17.464 ± 8.702	3.75 ± 0.526	0.083 ± 0.028
		F	9	9	1 ± 0.052	0.009 ± 0.004	6.833 ± 3.553	5.125 ± 0.398	0.3 ± 0.066
		M + F	17	16	0.993 ± 0.023	0.015 ± 0.008	11.838 ± 5.639	4.438 ± 0.365	0.192 ± 0.045
**All**	**15**	M + F	**87**	**29**	**0.934 ± 0.012**	**0.007 ± 0.005**	**3.1 ± 1.626**	**4.875 ± 0.273**	**0.239 ± 0.023**

*Note:* The table displays apiary name, number of colonies, sex (F = female, M = male), sample sizes (*N*) of small hive beetles, *Aethina tumida*, mitochondrial DNA diversity indices based on the COI gene (*h* = number of haplotypes, *H*_*d*_ = haplotype diversity, *P*_*i*_ = nucleotide diversity, and *K* = average number of nucleotide differences) and nuclear DNA diversity indices, *N*_*A*_ = number of alleles and *H*_*O*_ = observed heterozygosity. Bold values highlight the overall data for all the study sites.

### 2.2 DNA Extraction

Total DNA was extracted from whole individuals (three male and three female SHB from each of three colonies per apiary, *N* = 90) using a NucleoSpin Tissue kit (Macherey‐Nagel, Düren, Germany) following the manufacturer's instructions. DNA quantity and quality were assessed using a Quickdrop Spectrophotometer (Molecular Devices, San Jose, California, USA).

### 2.3 Mitochondrial DNA Haplotype Analysis

To identify and compare haplotypes within and across apiaries, DNA was amplified using COI barcoding primers AT1904S (5′‐GGTGGATCTTCAGTTGATTTAGC‐3′) and AT2953A (5′‐TCAGCTGGGGGATAAAATTG‐3′) (Evans et al. 2000). PCR was then conducted in a total reaction volume of 10 μL consisting of 1 μL DNA template, 6.55 μL of Millipore water, 2 μL of 5× My *Taq* reaction buffer (Meridian BioSciences, Memphis, Tennessee, USA), 0.2 μL of 10 μM forward and reverse primer, and 0.05 μL *Taq* polymerase (Meridian BioSciences, Memphis, Tennessee, USA) using a Biometra Thermal Cycler (Analytik Jena, Jena, Germany). The thermal conditions used were 95°C for 2 min, 40 cycles of 95°C for 20 s, 56°C for 30 s, 72°C for 30 s, and 72°C for 2 min. The PCR reactions included negative controls (Millipore water). The quality of the PCR products was verified on a 2% (w/v) agarose gel. The amplicons (1 μL) were diluted in 14 μL of Millipore water and sent to Microsynth AG (Balgach, Switzerland) for purification and Sanger sequencing, performed unidirectionally, using the forward primer.

The COI sequences were quality‐checked and trimmed in Chromas 2.5.0 (Technelysium Pty Ltd., 2018) to remove low‐quality bases and correct any ambiguous nucleotides. The resulting 787 bp sequences were aligned using the Muscle program in MEGA 11 (Tamura et al. 2021). Three of the sequences had poor‐quality electropherograms and were therefore removed. Additional SHB sequences (*N* = 69) from other areas of sub‐Saharan Africa (Idrissou et al. 2019) were included in the alignment (see Appendix Table S1). A maximum likelihood test was applied to identify the best evolutionary model in MEGA11 based on the Bayesian Information Criterion (BIC). The settings selected were partial deletion option and a site coverage cutoff of 95%. The best model, Tamura‐3‐parameter + Gamma distribution (T92 + G), was subsequently used to construct a maximum likelihood phylogenetic tree in MEGA 11 (Tamura et al. 2021).

The mtDNA haplotypes were determined using DnaSP v.6 (Librado and Rozas 2009), and a haplotype network tree, Templeton, Crandall, and Sing (TCS) tree (Templeton et al. 1992), was generated using the PopArt software (Leigh and Bryant 2015). Based on the clustering pattern in the network tree, the haplotypes were further classified into haplogroups. The pairwise genetic distances between sequences from the apiaries and haplogroups were calculated using the p‐distance method in MEGA 11. In this and all subsequent tests, normality was assessed using the Shapiro‐Wilks test in R v. 4.4.2 (R Core Team 2024), and in case normality was violated, a non‐parametric Kruskal‐Wallis test was used. Results were assumed statistically significant at a confidence level of *p* < 0.05.

The mtDNA indices, including the number of haplotypes (*h*), haplotype diversity (*H*_*d*_), nucleotide diversity (*P*_*i*_), and the average number of nucleotide differences (*K*), were determined using DnaSP v.6. These indices were compared within and across apiaries using a linear mixed model, with apiary as the fixed factor and SHB sex nested within colony as random factors. To compare the mtDNA indices between SHB males and females, a second linear mixed model was used, with SHB sex as the fixed factor and colony nested within apiary as random factors. The statistical analyses were conducted using the lme4 package (Bates et al. 2015) within R v. 4.4.2. Finally, the population structure of male and female SHB across the apiaries was assessed based on the mtDNA marker. The *F*_ST_ values for both sexes were calculated separately using DnaSP and then compared using a *t*‐test in R.

### 2.4 Nuclear DNA Diversity and Population Structure

To identify and compare nuDNA diversity and population structure within and across apiaries, DNA from 90 individuals was amplified using eight polymorphic DNA microsatellite loci, genotyped, and analyzed. The microsatellite primers used in the study were B89 (Evans et al. 2008); Atu‐8, Atu‐14, Atu‐16a, Atu‐17, Atu‐18 (Papach et al. 2022), and Atu‐9 and Atu‐11, which were designed for the current study (see Appendix Table S2). The microsatellite primers were tested on previously collected SHB samples from Kenya and were confirmed to be polymorphic. The PCR involved a total reaction volume of 10 μL composed of 1 μL DNA template, 6.55 μL of water, 2 μL of 5× My *Taq* reaction buffer (Meridian BioSciences, Memphis, Tennessee, USA), and 0.2 μL (10 μM) of each primer (forward primer and reverse primer; Microsynth AG Company, Balgach, Switzerland; see Appendix Table S2). It was conducted in a Biometra Thermal Cycler (Analytik Jena, Jena, Germany) under the following conditions: 95°C for 3 min, 35 cycles of 95°C for 30 s, 60°C for 30 s, 72°C for 30 s, and 72°C for 5 min. The PCR included both negative (Millipore water) and positive controls. The DNA was then sent to Microsynth AG (Balgach, Switzerland) for genotyping.

The microsatellite alleles were manually scored using the Peak Scanner v. 1.0 (Applied Biosystem, California, USA). The Microsatellite toolkit add‐in for Microsoft Excel (Park 2008) generated an input file that was used to create PCA plots of the SHB individuals using the *adegenet* package (Jombart 2008) in R v. 4.4.2. Linkage disequilibrium and Hardy Weinberg (H‐W) equilibrium tests were performed using Genepop v. 4.7.5 (Raymond and Rousset 1995; Rousset 2008). First, Fisher's test was used to assess linkage disequilibrium between pairs of markers within and across the eight microsatellite loci, confirming whether the markers were statistically independent. This involved 1000 dememorizations, 100 batches, and 1000 iterations per batch. Secondly, an H‐W test was used to determine whether the five apiary subpopulations alongside the eight loci were at H‐W equilibrium. A Bonferroni correction was applied with a significance level of *p* = 0.0018 for the linkage disequilibrium test and *p* = 0.01 and *p* = 0.006 for the H‐W test (population and loci, respectively). The non‐detection error was assessed for the microsatellite loci (Human et al. 2013). The presence of null alleles was determined in MicroChecker software v 2.2.3 (Van Oosterhout et al. 2004).

To determine the SHB genetic diversity levels within and across the five apiaries, the number of alleles per marker (*N*_*A*_) and observed heterozygosity (*H*_*O*_) were calculated using GenAlEx software version 6.5 (Peakall and Smouse 2012). These genetic indices were compared within and across apiaries using a linear mixed model, with apiary as the fixed factor and SHB sex nested within colony as random factors. A second linear mixed model was used to account for the effect of SHB sex on the nuclear indices, with SHB sex as the fixed factor and colony nested within the apiary as a random factor. The statistical analyses were conducted within R v. 4.4.2 using the lme4 package (Bates et al. 2015). In addition, a rarefaction analysis was performed using the ADZE software to assess whether the sample size was sufficient to capture the allelic richness of each apiary population (Szpiech et al. 2008).

Based on unexpectedly low heterozygosity levels in males, the physical locations of the microsatellite markers (*N* = 8) on chromosomes were ascertained. SHB has an XY sex‐determination system, composed of chromosome 1 (X chromosome), chromosomes 2–7 (autosomal), and chromosome 8 (male Y chromosome) (Huang et al. 2023). The forward sequence of each primer and its corresponding microsatellite region were compared against all eight chromosomes using the NCBI BLAST‐N database (Sayers et al. 2024). Seven markers were located on the sex chromosome 1, while the remaining locus, B89, was located on chromosome 3 (see Appendix Table S3). Thus, for subsequent analyses, SHB males had to be considered haploid at these seven loci. The genetic differentiation levels of male and female SHB were estimated using the HierFstat package in R (de Meeûs and Goudet 2007) across the seven X‐located loci because this package includes a function that considers diploid and haploid loci, unlike other classical *F*‐statistics methods. The global *F*‐statistics for males and females were calculated separately to generate two *F*‐statistics: *F*_colony/apiary_, assessing genetic differentiation at the colony level, and *F*_apiary/total_, assessing genetic differentiation at the apiary level relative to all populations. Both the *F*‐statistics and the *p*‐values were obtained after performing 1000 permutations. Finally, 1000 bootstraps were performed to obtain 95% confidence intervals. Sex‐bias dispersal can result in different levels of genetic differentiation between males and females. These differences are considered statistically significant when the confidence intervals around F‐statistic estimates are non‐overlapping (Mossmann and Wasser 2002). Isolation by distance was determined by comparing the genetic distance Dest/(1 − Dest) with the natural log (In) geographical distance (in km) across the apiaries (Wright 1946) using GenAlEx software version 6.5 (Peakall and Smouse 2012).

## 3 Results

### 3.1 Mitochondrial DNA Haplotypes

Among the 87 SHB individuals analyzed, 29 haplotypes were identified (Table 1). These haplotypes were further grouped into haplogroups A and B (*p*‐distance = 5.6%). With one exception, all individuals belonged to haplogroup A, and the haplotypes were randomly distributed across the apiaries (Kruskal‐Wallis, *χ* ^2^ = 3.733, *p* > 0.05; Figure 2). Haplogroup B consisted only of a single individual (Figure 2). The range of pairwise genetic distances within and across the apiaries was 0.7%–1.5% and 0.6%–1.2%, respectively (Table 2). Previously published SHB samples (Idrissou et al. 2019) from other regions in sub‐Saharan African region clustered seemingly randomly with either Haplogroup A or Haplogroup B individuals (Figure 3).

**FIGURE 2:**
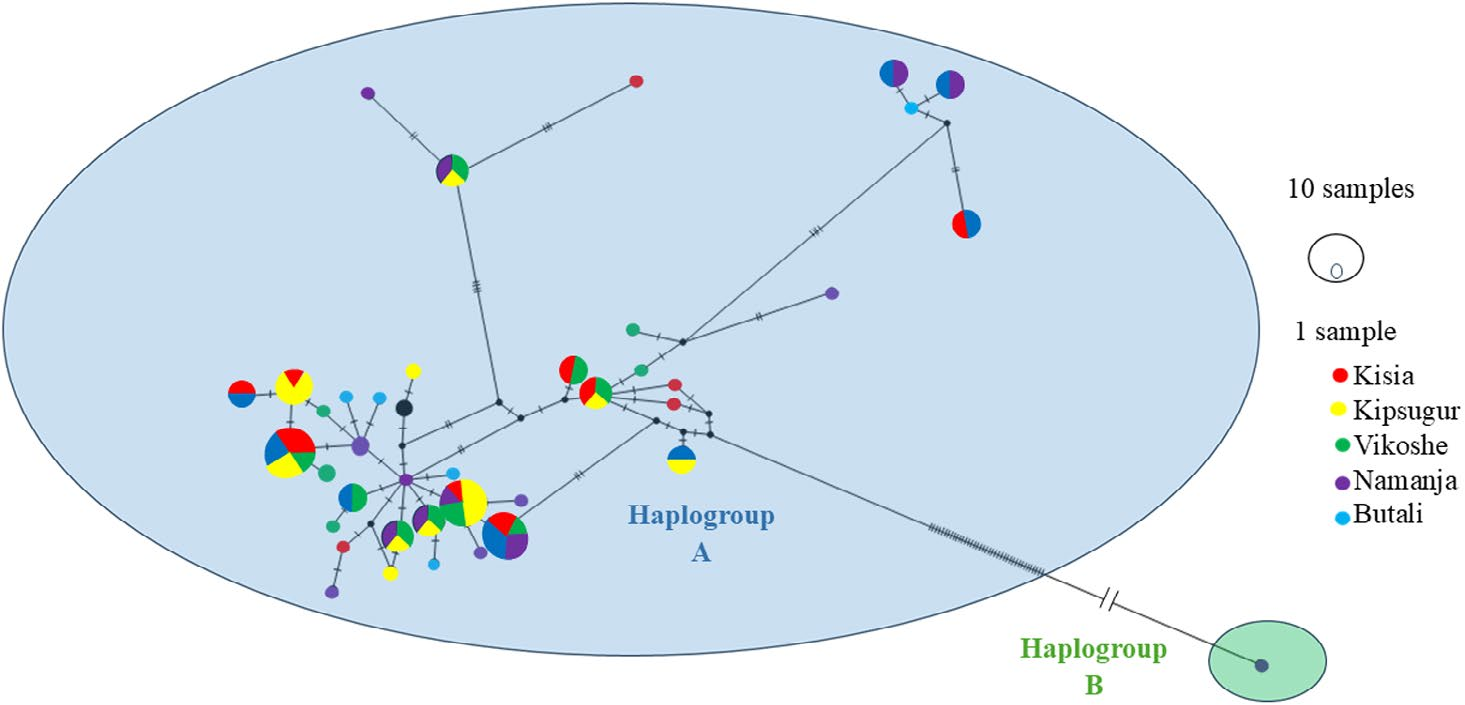
Haplotype network tree for small hive beetles. Mitochondrial haplotype network of small hive beetles, *Aethina tumida* , at five apiaries in Kakamega County, Western Kenya. The large ellipses represent the haplogroups: blue = Haplogroup A and green = Haplogroup B. The pie charts represent the distribution of SHB haplotypes based on the apiary sites. Red = Kisia, yellow = Kipsugur, green = Vikoshe, blue = Namanja, and purple = Butali. Small ticks on branches indicate the number of mutations separating haplotypes.

**TABLE 2:** Genetic distances between and within apiaries based on mitochondrial DNA.

Comparison	Apiaries				
	Kisia	Kipsugur	Vikoshe	Namanja	Butali
Between Apiary variation					
Kipsugur	0.006 ± 0.001				
Vikoshe	0.006 ± 0.001	0.006 ± 0.001			
Namanja	0.007 ± 0.002	0.007 ± 0.001	0.007 ± 0.001		
Butali	0.011 ± 0.002	0.011 ± 0.002	0.011 ± 0.002	0.012 ± 0.002	
Within Apiary variation	0.007 ± 0.002	0.005 ± 0.001	0.006 ± 0.002	0.008 ± 0.002	0.015 ± 0.002

*Note:* The table shows mean genetic distances ± standard deviations for small hive beetles, *Aethina tumida*, based on mitochondrial DNA within and across five apiaries (Kisia, Kipsugur, Vikoshe, Namanja, and Butali) in the Kakamega region, Western Kenya.

**FIGURE 3:**
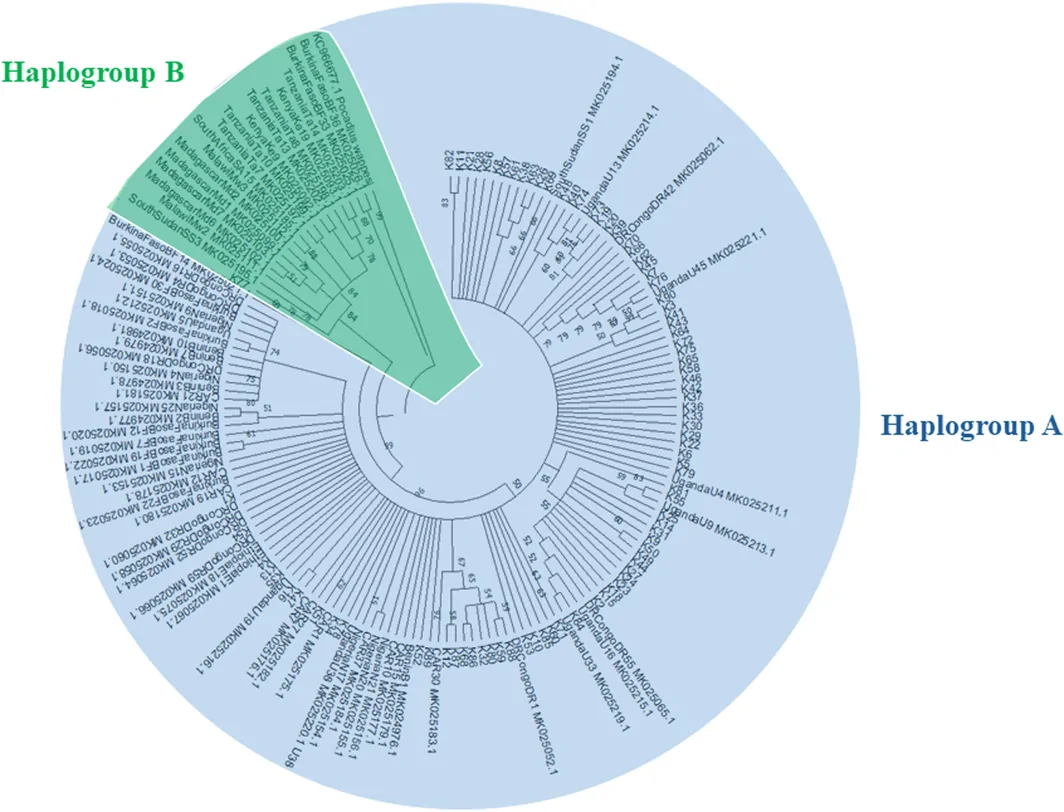
Phylogenetic tree for small hive beetles in their native range. Phylogenetic tree of the small hive beetle, *Aethina tumida*, COI gene sequences (*n* = 87) sampled from five apiaries in Kakamega County, Western Kenya, and published sequences from other sub‐Saharan countries. The samples with an NCBI accession number are from Idrissou et al. (2019), while samples abbreviated as K are new sequences from this study. K1–K18 = Apiary 1 (Kisia), K19–K36 = Apiary 2 (Kipsugur), K37–K54 = Apiary 3 (Vikoshe), K55–K72 = Apiary 4 (Namanja) and K73–K90 = Apiary 5 (Butali). The blue portion represents Haplogroup A, and the green portion represents Haplogroup B.

No significant differences across apiaries were found in terms of mtDNA diversity indices (linear mixed model, *h*: *F* = 0.886, *p* > 0.05; *H*_*d*_: *F* = 0.334, *p* > 0.05; *P*_*i*_: *F* = 0.702, *p* > 0.05; *K*: *F* = 0.738, *p* > 0.05). There were also no significant differences in mtDNA diversity indexes between SHB males and females from the different apiaries and colonies (linear mixed model, *h*: *F* = 3.5, *p* > 0.05; *H*_*d*_: *F* = 2.744, *p* > 0.05; *P*_*i*_: *F* = 0.231, *p* > 0.05; *K*: *F* = 0.309, *p* > 0.05). The male and female *F*_ST_ values across the apiaries were low and nonsignificant (*t*‐test, *t* = −0.4061, *p* > 0.05; Table 2).

### 3.2 Nuclear DNA Diversity and Population Structure

A total of 90 genotyped individuals were used for the nuDNA analysis. The percentage of missing data in the final file was 2.27% for males and 1.38% for females. The presence of null alleles was confirmed in all the loci for the diploid female data (see Appendix Table S4). The males were not inspected for null alleles as the transformation of haploid data into homozygous diploid data would violate the software's assumptions. However, the low frequency of missing alleles in the male data file suggests that null alleles played a minor role in the subsequent analyses.

None of the loci pair combinations were in linkage disequilibrium after Bonferroni correction (*p* > 0.0018). Furthermore, the different populations (*p* < 0.01) and loci (*p* < 0.006) deviated from H‐W equilibrium after Bonferroni correction. The overall non‐detection error for all loci was 0.05%. The rarefaction analysis for both males (*N* = 44) and females revealed that the sample size (*N* = 46) was sufficient to capture the allelic diversity (see Appendix Figure S1).

No significant differences across apiaries were found for the number of alleles per marker (*N*_*A*_) and observed heterozygosity (*H*_*O*_) (linear mixed model, *F* = 1.024, *p* > 0.05; *F* = 1.979, *p* > 0.05, *N*_*A*_ and *H*_*O*_, respectively; Figure 4a,b). However, there was a significant difference in these indices between SHB males and females from the different apiaries (*F* = 48.251, *p* < 0.0001; *F* = 293.487, *p* < 0.0001, *N*_*A*_ and *H*_*O*_, respectively; Figure 4a,b and Table 3a,b).

**FIGURE 4:**
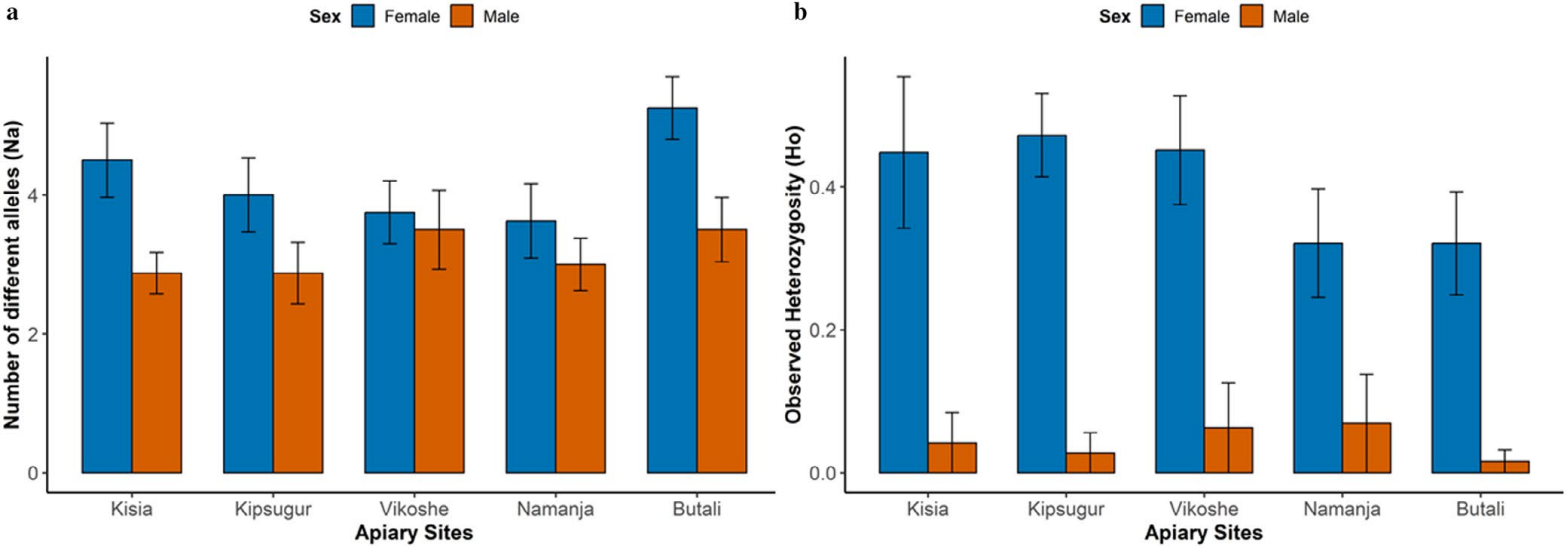
Nuclear DNA diversity across apiaries and sexes. Bar graphs showing (a) the average number of different alleles (*N*_*A*_, ±SE) and (b) average observed heterozygosity (*H*_*O*_, ±SE) over all markers of small hive beetles, *Aethina tumida* , from five different apiary locations in Western Kenya based on X‐located markers (*N* = 7) and the autosomal marker (*N* = 1). Although male SHB were considered haploid at the X‐linked loci, they had some heterozygous individuals at the autosomal marker, resulting in a low observed heterozygosity rather than null heterozygosity. The linear model shows a significant difference between male and female SHB from the different apiaries (*F* = 48.251, *p* < 0.0001; *F* = 293.487, *p* < 0.0001, *N*_*A*_ and *H*_*O*_, respectively).

**TABLE 3:** Genetic distances between and within apiaries based on nuclear DNA.

(a)				
Males	Kisia	Kipsugur	Vikoshe	Namanja
Kipsugur	0.026			
Vikoshe	0.035	0.026		
Namanja	0.046	0.052	0.045	
Butali	0.040	0.031	0.043	0.042

(b)				
Females	Kisia	Kipsugur	Vikoshe	Namanja
Kipsugur	0.047			
Vikoshe	0.048	0.035		
Namanja	0.072	0.036	0.054	
Butali	0.066	0.046	0.037	0.045

*Note:* Tables showing the pairwise genetic distance for male (a) and female (b) small hive beetles, *Aethina tumida*, based on nuclear DNA across five apiaries (Kisia, Kipsugur, Vikoshe, Namanja, Butali) in the Kakamega region, Western Kenya.

The PCA analyses showed high admixture in both males and females (Figure 5a,b). However, SHB males showed a lower differentiation across apiaries (*F*_apiary/total_ = −0.049), compared to females (*F*_apiary/total_ = 0.012), with non‐overlapping 95% confidence intervals (males: −0.064 to −0.029; females: −0.005 to 0.030), implying a statistically significant difference. On the other hand, SHB females showed a lower differentiation between colonies of the same apiaries (*F*_colony/apiary_ = −0.026), compared to males (*F*_colony/apiary_ = 0.091), with non‐overlapping 95% confidence intervals (females: −0.074 to 0.021; males: 0.036 to 0.136), implying a statistically significant difference.

**FIGURE 5:**
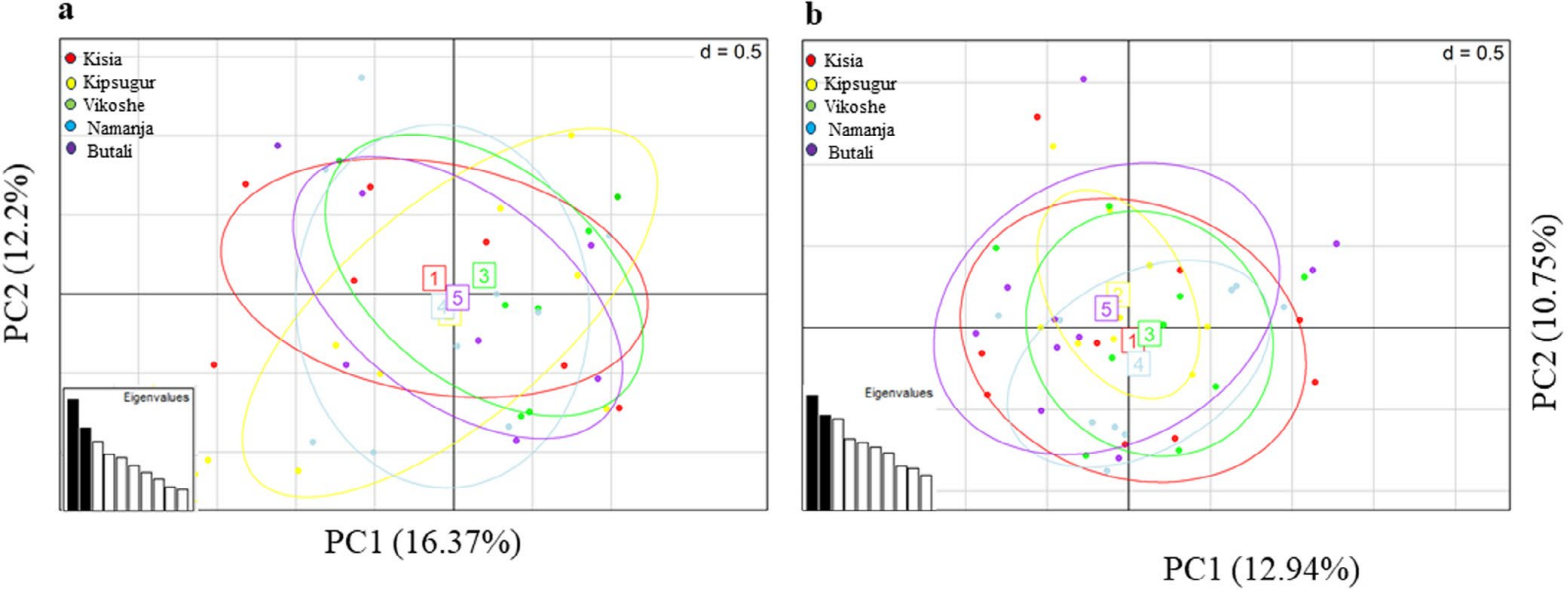
Principal Component Analysis (PCA) plot of DNA microsatellite sequences of small hive beetles, *Aethina tumida*, from five apiaries in Western Kenya. PCA plots of (a) male and (b) female small hive beetles, *Aethina tumida* , with ellipses, dots, and middle squares colored to distinguish the different apiary sites as red = Kisia (1), yellow = Kipsugur (2), green = Vikoshe (3), blue = Namanja (4), and purple = Butali (5). The ellipses show the distribution of SHB individuals sampled from the different apiary sites represented as dots. The PC1 values on the *x*‐axis and PC2 on the *y*‐axis represent the first and second principal components explaining the largest variance.

The correlation between the genetic distance and geographical distance (i.e., isolation by distance, IBD) was significant for females (*F* = 5.64, *p* = 0.044) but not for males (*F* = 0.44, *p* = 0.529; Figure 6). Moreover, the correlation yielded a less steep slope for males (*y* = 0.0055*X*—0.0349, *R* ^2^ = 0.0363) than for females (*y* = 0.0105*X*—0.0218, *R* ^2^ = 68; Figure 6).

**FIGURE 6:**
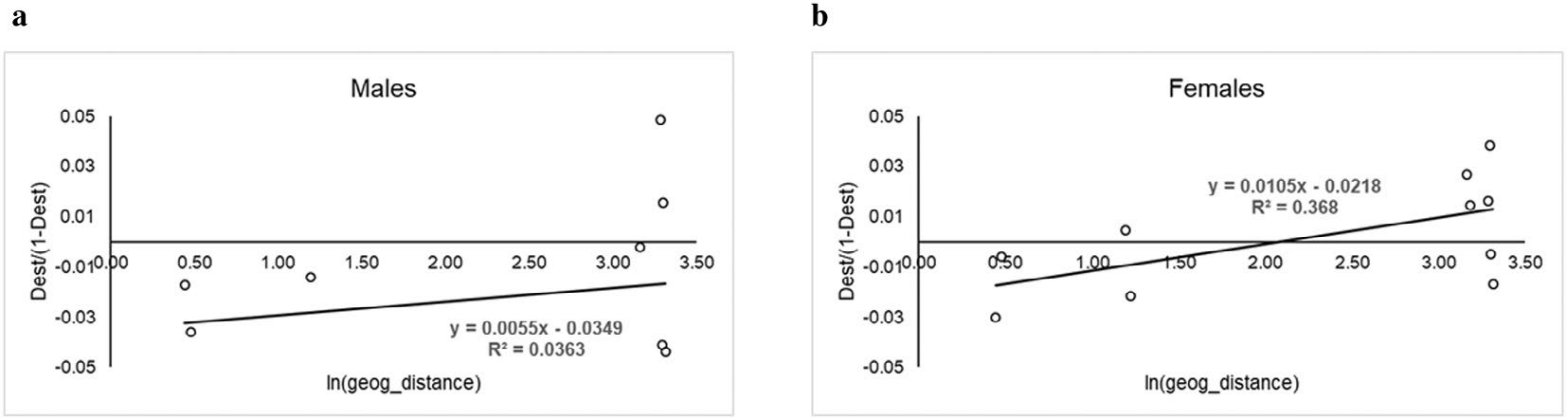
Isolation by distance plots. Graphs showing the correlation between the pairwise genetic distance (Dest/(1 − Dest)) and geographical distance (ln (km)) across the five apiaries for both males (a) and females (b). The regression equations, their *R* ^2^ and the *p*‐values are also provided.

## 4 Discussion

In this study, we investigated the genetic diversity and dispersal of SHB ( *A. tumida* ) between apiaries in its native range. High levels of mtDNA and nuDNA genetic diversity, as well as a homogenous genetic structure, were found when comparing SHB within and across apiaries, suggesting high dispersal at the level considered. Moreover, SHB males displayed lower levels of genetic differentiation than females across apiaries, suggesting that SHB males disperse more than females.

The comparison of haplotype diversity and distribution through mtDNA analysis revealed a high level of diversity in the region which is part of the native range. Nearly all the haplotypes identified in the study clustered within haplogroup A, one of the two SHB clades that have been described globally so far (Cordeiro et al. 2019; Idrissou et al. 2019). Haplogroup A consists of SHB from Central, Western, and Eastern Africa and is more heterogeneous than the second haplogroup, B consisting of SHB from Eastern, Southern, and Western Africa. Haplogroup A is also considered the possible origin of SHB (Idrissou et al. 2019). In contrast, the invasive range has far fewer haplotypes and thus a lower mtDNA diversity. Notably, two widely dispersed haplotypes were identified in the USA (NA1 and NA2) while the Brazilian and South Korean populations, each had a single haplotype. These haplotypes all belong to Haplogroup B (Evans et al. 2000, 2003, 2008; Lounsberry et al. 2010; Cordeiro et al. 2019; Namin et al. 2019). The mtDNA results also revealed a pattern of high gene flow over short‐ and medium‐range distances (within and across apiaries, respectively). These results align with previous studies showing high gene flow on a broader scale (Evans et al. 2000; Lounsberry et al. 2010; Idrissou et al. 2019). At the country level, two South African samples separated by hundreds of kilometers, shared identical haplotypes (Evans et al. 2000). At an even broader continental scale, SHB from Burkina Faso in Western Africa clustered with South African beetles (Idrissou et al. 2019). Taken together, the high mtDNA gene flow at both small and large scale levels could be a result of combination of SHB flight dispersal and human‐mediated dispersal through migratory beekeeping, resulting in the co‐occurrence of distinct haplotypes and haplogroups in the same apiaries and even colonies (Idrissou et al. 2019; Cornelissen et al. 2024). The findings also suggest that lineages may spread both naturally and by human transportation widely within the native range (Lounsberry et al. 2010).

In parallel with the mtDNA analysis, microsatellite markers were also used to compare the nuDNA diversity and differentiation levels within and across apiaries. Similar to the mtDNA profile, the nuDNA diversity levels did not differ within and across the apiaries, confirming high gene flow. Both nuDNA diversity indices, *N*_*A*_ and *H*_*O*_, were higher in females than in males because the seven loci used in the study were located on the X‐chromosome. The SHB X chromosome is twice as long in females as in males (Huang et al. 2023), so females may have a higher nuDNA diversity than males on this chromosome. Diversity variation between males and females on the X‐chromosome has also been observed in *Drosophila melanogaster* . In males, the expression of genes with deleterious or slightly deleterious effects is unmasked, resulting in a reduction of allelic variation, while in females, any deleterious alleles can be masked by the second X‐chromosome (Stocks et al. 2015). Additionally, X‐linked genes spend more time in females than in males and may acquire more genes with female functions or sexually antagonistic effects, which increases their genetic variation (Parker et al. 2022). This may apply to SHB as well, as both sexes are diploid for the autosomes, but not on the X chromosome (Huang et al. 2023).

Additionally, the nuDNA differentiation of SHB females within apiaries was lower than that of males. Conversely, the nuDNA differentiation of SHB males between apiaries was lower than that of females. The IBD analysis also revealed sex‐specific patterns, with a statistically significant association between geographical and genetic distances and a stronger effect in females than in males. These results may reflect male‐biased dispersal over long distances, confirming previous work that has reported differences in dispersal behavior between males and females. SHB males tend to fly earlier than females (Elzen et al. 2000), while SHB females tend to fly upwind towards colonies (Cornelissen et al. 2024). Furthermore, SHB females have been shown to employ a sit‐and‐wait strategy in finding their mates (Neumann et al. 2016), and SHB males mate with multiple females (Papach et al. 2022). Taken together, these studies suggest male‐biased dispersal for SHB. However, since the microsatellite markers used in the study were located on the X chromosome, the results on male‐biased dispersal should be interpreted with caution, and confirmation with autosomal markers would be worthwhile. In several insect species, male‐biased dispersal has been reported. It is favored by a smaller body size in the male forest ground beetle *Pterostichus oblongopunctatus* than females (Lagisz et al. 2010); a high flight performance in the male *Elater ferrugineus* beetle (Martínez‐Pérez et al. 2022), and a lower mobility of females Taylor's checkerspot butterfly after locating a suitable, nutrient‐rich oviposition site (Bennett et al. 2013). If confirmed in SHB, male‐biased dispersal could help avoid inbreeding and reduce competition with relatives (Perrin and Mazalov 2000).

Overall, the high gene flow in both mtDNA and nuDNA found in this study occurred irrespective of the geographical distance separating the SHB sampled, and genetic distance was similar when comparing the closest apiaries (2 km apart) and farthest apiaries (30 km apart). This high gene flow pattern can be related to SHB dispersal that occurs by (i) SHBs flying alone (Cornelissen et al. 2024), (ii) flying in swarms of conspecifics (Tribe 2000) in search of hosts and mates (Cornelissen et al. 2024), (iii) joining honeybee swarms (Hepburn and Radloff 1998; Spiewok et al. 2008). The fourth possible mechanism, migratory beekeeping, could have a little or no influence on the gene flow, as apiaries used in the study were from stationary beekeepers. Furthermore, migratory beekeeping is infrequently practiced in sub‐Saharan Africa, except in South Africa (Nganso et al. 2025). The contributions of these mechanisms to the short and medium‐range distances studied here remain unclear and it would be worthwhile investigating their importance to SHB dispersal.

## 5 Conclusions

This study reveals how SHB, an invasive pest, exhibits high gene flow patterns (high dispersal) and apparently no genetic structure at a fine‐spatial scale (across and within apiaries) in its native range. This pattern is probably due to a combination of SHB's inherent flight capabilities, association with its honeybee host, and human‐mediated transport enhancing its spread over long distances. The results reported here suggest that males may disperse over longer distances than females. Establishing this fully could benefit future mitigation measures, such as the development of sex pheromone traps that target only males and suppress their long‐distance dispersal. As dispersal is a trait that is inherently difficult to characterize, studies combining population genetics and direct measures of dispersal are needed to strengthen current management efforts to contain the spread of invasive pests.

## Author Contributions

**Michael N. K. Muturi:** conceptualization (supporting), formal analysis (lead), investigation (equal), software (lead), visualization (equal), writing – original draft (lead). **H. Michael G. Lattorff:** conceptualization (supporting), formal analysis (equal), investigation (equal), methodology (equal), supervision (equal), validation (lead), visualization (equal), writing – review and editing (equal). **Nkoba Kiatoko:** methodology (equal), project administration (supporting), resources (equal), writing – review and editing (equal). **Peter Neumann:** conceptualization (equal), formal analysis (supporting), funding acquisition (lead), investigation (equal), methodology (equal), project administration (lead), resources (lead), supervision (equal), writing – review and editing (equal). **Alexis Beaurepaire:** conceptualization (equal), formal analysis (equal), funding acquisition (supporting), investigation (lead), methodology (lead), project administration (supporting), software (supporting), supervision (lead), visualization (equal), writing – original draft (supporting), writing – review and editing (lead).

## Funding

This work was supported by Federal Commission for Scholarships for Foreign Students, 2022.0520, Vinetum Foundation, Schweizerischer Nationalfonds zur Förderung der Wissenschaftlichen Forschung, 310030_204479.

## Conflicts of Interest

The authors declare no conflicts of interest.

## Supporting information


**Figure S1:** Rarefaction analysis of small hive beetle male and female populations. Line graphs showing the mean number of distinct alleles per locus against the standardized sample size for both female (blue line) and male (orange line) small hive beetle, *Aethina tumida* , subpopulations from five apiary locations in Western Kenya.
**Table S1:** Small hive beetle sequences. Published small hive beetle sequences (Idrissou et al. 2019) from Sub‐Saharan Africa that were included in the study (see Figure 3). The sequence ID, their respective accession numbers and the haplogroups of the sequences are shown.
**Table S2:** Primer information. List of primers used for the PCR reactions. Marker name, Sequence 5″–3″, size, repeat motif, number of different alleles, and references are shown.
**Table S3:** Loci information. Position of the Forward‐primer sequence and PCR product region on Chromosome 1 of *Aethina tumida* CM044479.1 of seven of eight loci used in the study.
**Table S4:** Null alleles. Estimates of null allele frequencies of female SHB (*N* = 46) across the microsatellite loci. Oosterhout, Chakraborty, Brookfield1 and Brookfield 2 represent the estimators used in MICROCHECKER software to calculate the null frequencies.

## Data Availability

Genotype raw data are deposited on the Bern Open Repository and Information System (BORIS) under the address (https://doi.org/10.48620/101752).
