# Supplementary material for: Population Genetics of Small Hive Beetles in Their Native Range Indicates High Gene Flow and Male‐Biased Dispersal
Source: Ecol Evol. 2026 Nov 10;16(11):e74668. doi: 10.1002/ece3.74668 (PMC13751221; doi:10.1002/ece3.74668)
Supplement: Supplementary file 1 [file ECE3-16-e74668-s001.docx]

**Appendix**

**Figure S1: Rarefaction analysis of small hive beetle male and female populations**

Line graphs showing the mean number of distinct alleles per locus against the standardized sample size for both female (blue line) and male (orange line) small hive beetle, *Aethina tumida*, subpopulations from five apiary locations in Western Kenya.

**Table S1:** **Small hive beetle sequences**

Published small hive beetle sequences (Iddrisou et al 2019) from Sub-Saharan Africa that were included in the study (see Figure 3). The sequence ID, their respective accession numbers and the haplogroups of the sequences are shown.

| **Sequence ID** | **Accession number** | **Haplogroup** |
| --- | --- | --- |
| BeninB1 | MK024976.1 | A |
| BeninB2 | MK024977.1 | A |
| BeninB3 | MK024978.1 | A |
| BeninB7 | MK024979.1 | A |
| BeninB10 | MK024981.1 | A |
| BurkinaFasoBF1 | MK025017.1 | A |
| BurkinaFasoBF2 | MK025018.1 | A |
| BurkinaFasoBF7 | MK025019.1 | A |
| BurkinaFasoBF12 | MK025020.1 | A |
| BurkinaFasoBF14 | MK025021.1 | A |
| BurkinaFasoBF19 | MK025022.1 | A |
| BurkinaFasoBF22 | MK025023.1 | A |
| BurkinaFasoBF30 | MK025024.1 | A |
| BurkinaFasoBF33 | MK025025.1 | B |
| BurkinaFasoBF36 | MK025026.1 | B |
| DRCongoDR1 | MK025052.1 | A |
| DRCongoDR4 | MK025053.1 | A |
| DRCongoDR16 | MK025055.1 | A |
| DRCongoDR18 | MK025056.1 | A |
| DRCongoDR29 | MK025058.1 | A |
| DRCongoDR32 | MK025060.1 | A |
| DRCongoDR42 | MK025062.1 | A |
| DRCongoDR52 | MK025064.1 | A |
| DRCongoDR55 | MK025065.1 | A |
| DRCongoDR59 | MK025066.1 | A |
| EthiopiaE1 | MK025067.1 | A |
| EthiopiaE18 | MK025075.1 | A |
| KenyaKe9 | MK025092.1 | B |
| KenyaKe19 | MK025093.1 | B |
| MadagascarMd1 | MK025099.1 | B |
| MadagascarMd2 | MK025100.1 | B |
| MadagascarMd6 | MK025102.1 | B |
| MadagascarMd7 | MK025103.1 | B |
| MalawiMw2 | MK025111.1 | B |
| MalawiMw3 | MK025112.1 | B |
| NigeriaN4 | MK025150.1 | A |
| NigeriaN9 | MK025151.1 | A |
| NigeriaN15 | MK025153.1 | A |
| NigeriaN17 | MK025154.1 | A |
| NigeriaN20 | MK025155.1 | A |
| NigeriaN21 | MK025156.1 | A |
| NigeriaN25 | MK025157.1 | A |
| CAR1 | MK025175.1 | A |
| CAR7 | MK025176.1 | A |
| CAR10 | MK025177.1 | A |
| CAR12 | MK025178.1 | A |
| CAR15 | MK025179.1 | A |
| CAR19 | MK025180.1 | A |
| CAR21 | MK025181.1 | A |
| CAR27 | MK025182.1 | A |
| CAR30 | MK025183.1 | A |
| CAR37 | MK025184.1 | A |
| SouthAfricaSA15 | MK025185.1 | B |
| SouthSudanSS1 | MK025194.1 | A |
| SouthSudanSS3 | MK025195.1 | B |
| TanzaniaTa7 | MK025197.1 | B |
| TanzaniaTa8 | MK025198.1 | B |
| TanzaniaTa10 | MK025199.1 | B |
| TanzaniaTa13 | MK025202.1 | B |
| TanzaniaTa14 | MK025203.1 | B |
| UgandaU4 | MK025211.1 | A |
| UgandaU5 | MK025212.1 | A |
| UgandaU9 | MK025213.1 | A |
| UgandaU13 | MK025214.1 | A |
| UgandaU16 | MK025215.1 | A |
| UgandaU19 | MK025216.1 | A |
| UgandaU33 | MK025219.1 | A |
| UgandaU38 | MK025220.1 | A |
| UgandaU45 | MK025221.1 | A |

**Table S2: Primer information**

List of primers used for the PCR reactions. Marker name, Sequence 5”–3”, size, repeat motif, number of different alleles, and references are shown.

| **Marker** | **Sequence 5”–3”** | **Size (bp)** | **Repeat motif** | **Number of different alleles** | **Reference** |
| --- | --- | --- | --- | --- | --- |
| Atu-8 | ATGCCAATGCAACAACGTAA | 296 | Tri | 8 | Papach et al*.* 2023 |
|  | TCCTGAACAGTTTCCTGACCT |  |  |  |  |
| Atu-9 | TCACGACGGCAAGTTTAACA | 287 | Di | 11 | Current study |
|  | AAACCGTGGACCGTTAATTG |  |  |  |  |
| Atu-11 | CGGCGTCTTGTGATGATTTA | 281 | Di | 10 |  |
|  | AAAAACAAACACGCGAATCC |  |  |  |  |
| Atu-14 | CTTACACATTTCGGGCCATT | 217 | Tri | 17 | Papach et al*.* 2023 |
|  | CCCTTCTCTCAATAGATTGCTTT |  |  |  |  |
| Atu-16a | CGCCGTTAACACGTAACACA | 118 | Tri | 15 |  |
|  | AGCGACCGCAGTTACTGAAT |  |  |  |  |
| Atu-17 | CATGATGGTTCGTGAGCAAC | 168 | Tri | 10 |  |
|  | TGGCCACTATAACAGCCAAT |  |  |  |  |
| Atu-18a | AATACGCATGTTTGCCACTTC | 191 | Di | 12 |  |
|  | GGGTTTGGCCATCTGAAAAT |  |  |  |  |
| B89 | CGTCGCTATTAGCCCCTTTA | 110 | Di | 8 | Evans et al. 2008 |
|  | CTGACCTTCTCGTGCAACAA |  |  |  |  |

**Table S3: Loci information**

Position of the Forward-primer sequence and PCR product region on Chromosome 1 of *Aethina tumida* CM044479.1 of seven of eight loci used in the study.

| **Loci ID** | **F-primer position (bp)** | **PCR amplicon position (bp)** |
| --- | --- | --- |
| Atu-8 | 17 031 672 to 17 031 691 | 17 031 501 to 17 032 270 |
| Atu-9 | 16 569 886 to 16 569 905 | 15 605 037 to 15 605 804 |
| Atu-11 | 16 522 151 to 16 522 170 | 16 521 959 to 16 522 726 |
| Atu-14 | 15 605 369 to 15605388 | 15 605 037 to 15 605 804 |
| Atu-16 | 16 554 849 to 16 554 868 | 16 554 526 to 16 555 295 |
| Atu-17 | 17 225 797 to 17 225 816 | 17 225 546 to 17 226 318 |
| Atu-18a | 15 173 044 to 15 173 064 | 15 172 748 to 15 173 519 |

**Table S4: Null alleles**

Estimates of null allele frequencies of female SHB (N =46) across the microsatellite loci. Oosterhout, Chakraborty, Brookfield1 and Brookfield 2 represent the estimators used in MICROCHECKER software to calculate the null frequencies.

| **Locus** | **Null Present** | **Oosterhout** | **Chakraborty** | **Brookfield 1** | **Brookfield 2** |
| --- | --- | --- | --- | --- | --- |
| Atu16 | no | 0.1618 | 0.1429 | 0.0571 | 0.302 |
| Atu18 | yes | 0.1826 | 0.2381 | 0.1515 | 0.1515 |
| Atu11 | yes | 0.2133 | 0.28 | 0.1931 | 0.3443 |
| Atu9 | no | 0.08 | 0.0943 | 0.0725 | 0.0725 |
| Atu8 | yes | 0.3446 | 1 | 0.2537 | 0.2537 |
| B89 | yes | 0.2926 | 0.4203 | 0.2507 | 0.2507 |
| Atu14 | yes | 0.1518 | 0.2032 | 0.1453 | 0.1453 |
| Atu17 | yes | 0.3729 | 0.7122 | 0.3103 | 0.3103 |
